# The Influence of Prebiotic Arabinoxylan Oligosaccharides on Microbiota Derived Uremic Retention Solutes in Patients with Chronic Kidney Disease: A Randomized Controlled Trial

**DOI:** 10.1371/journal.pone.0153893

**Published:** 2016-04-21

**Authors:** Ruben Poesen, Pieter Evenepoel, Henriette de Loor, Jan A. Delcour, Christophe M. Courtin, Dirk Kuypers, Patrick Augustijns, Kristin Verbeke, Björn Meijers

**Affiliations:** 1 Department of Microbiology and Immunology, Division of Nephrology, University Hospitals Leuven, B-3000, Leuven, Belgium; 2 Department of Microbial and Molecular Systems, Centre for Food and Microbial Technology, University of Leuven, B-3000, Leuven, Belgium; 3 Leuven Food Science and Nutrition Research Centre (LFoRCe), University of Leuven, B-3000, Leuven, Belgium; 4 Department of Pharmaceutical and Pharmacological Sciences, Drug Delivery and Disposition, University of Leuven, B-3000, Leuven, Belgium; 5 Translational Research for Gastrointestinal Disorders (Targid), University of Leuven, B-3000, Leuven, Belgium; Medical University of Graz, AUSTRIA

## Abstract

The colonic microbial metabolism is a key contributor to uremic retention solutes accumulating in patients with CKD, relating to adverse outcomes and insulin resistance. Whether prebiotics can reduce intestinal generation of these microbial metabolites and improve insulin resistance in CKD patients not yet on dialysis remains unknown. We performed a randomized, placebo-controlled, double-blind, cross-over study in 40 patients with eGFR between 15 and 45 ml/min/1.73 m^2^. Patients were randomized to sequential treatment with prebiotic arabinoxylan oligosaccharides (AXOS) (10 g twice daily) and maltodextrin for 4 weeks, or vice versa, with a 4-week wash-out period between both intervention periods. Serum levels and 24h urinary excretion of *p*-cresyl sulfate, *p*-cresyl glucuronide, indoxyl sulfate, trimethylamine N-oxide and phenylacetylglutamine were determined at each time point using liquid chromatography—tandem mass spectrometry. In addition, insulin resistance was estimated by the homeostatic model assessment (HOMA-IR). A total of 39 patients completed the study. We observed no significant effect of AXOS on serum *p*-cresyl sulfate (*P* 0.42), *p*-cresyl glucuronide (*P* 0.59), indoxyl sulfate (*P* 0.70) and phenylacetylglutamine (*P* 0.41) and a small, albeit significant decreasing effect on serum trimethylamine N-oxide (*P* 0.04). There were neither effect of AXOS on 24h urinary excretion of *p*-cresyl sulfate (*P* 0.31), *p*-cresyl glucuronide (*P* 0.23), indoxyl sulfate (*P* 0.87) and phenylacetylglutamine (*P* 0.43), nor on 24h urinary excretion of trimethylamine N-oxide (*P* 0.97). In addition, we observed no significant change in HOMA-IR (*P* 0.93). In conclusion, we could not demonstrate an influence of prebiotic AXOS on microbiota derived uremic retention solutes and insulin resistance in patients with CKD not yet on dialysis. Further study is necessary to elucidate whether prebiotic therapy with other characteristics, higher cumulative exposure or in different patient populations may be of benefit.

***Trial Registration***: Clinicaltrials.gov NCT02141815

## Introduction

The uremic milieu is consequential to a disrupted balance between availability of retention solutes and the excretory capacity of the kidney [1;2]. There is substantial evidence that the colonic microbial metabolism is a key contributor to uremic retention solutes accumulating in patients with chronic kidney disease (CKD) [3;4]. Until recently, research has mainly focused on *p*-cresyl sulfate (PCS) and indoxyl sulfate (IS). PCS originates from microbial fermentation of tyrosine to *p*-cresol with subsequent endogenous sulfation. Likewise, IS is derived from microbial fermentation of tryptophan to indole, followed by endogenous oxidation and sulfation [4]. Both PCS and IS have repeatedly been associated with overall mortality, cardiovascular disease and progression of pre-existing renal disease [5–10]. *p*-Cresyl glucuronide (PCG) is another *p*-cresol derivative formed by endogenous glucuronidation, also relating to worse survival in patients with renal dysfunction [11]. Several other microbiota derived uremic retention solutes have been identified, including trimethylamine N-oxide (TMAO) and phenylacetylglutamine (PAG), which are derived from microbial metabolism of choline and phenylalanine, respectively. Recent data in patients with CKD point to a relationship between cardiovascular disease and serum levels of both TMAO and PAG, while TMAO may also be involved in the development of renal disease [12–14].

Given its importance in uremia, the colonic microbial metabolism is considered a promising, albeit unconventional and largely unexplored therapeutic target in CKD. As reviewed elsewhere, various strategies may impact the colonic microbial metabolism, including prebiotics [15]. Prebiotics are generally defined as selectively fermented ingredients that result in specific changes in the composition and/or activity of the gastrointestinal microbiota (i.e., mainly bifidobacteria), thus conferring benefit(s) upon host health, which is mainly derived from a shift from protein fermentation to carbohydrate fermentation [16]. Previously, we have demonstrated a decreasing effect of prebiotic oligofructose-enriched inulin on serum levels of PCS, but not IS, in hemodialysis patients [17]. Recently, a reduction in serum levels of IS and, possibly, PCS in hemodialysis patients has also been observed with resistant starch [18].

Whether prebiotic therapy is beneficial in patients with CKD not yet on dialysis remains unknown. Only one preclinical animal study has explored the effect of prebiotics in moderate CKD [19]. In mice with normal renal function, it has been shown by Koppe *et al*. that PCS in uremic concentrations triggers insulin resistance, loss of fat mass and ectopic redistribution of lipid in muscle and liver, mimicking features observed in mice after induction of 5/6^th^ nephrectomy. Interestingly, when arabinoxylan oligosaccharides (AXOS), which has prebiotic characteristics as summarized by Broekaert *et al*. [20], was administered to 5/6^th^ nephrectomy mice, these metabolic derangements were reversed with a concomitant reduction in serum PCS. Our research groups have previously performed several studies with AXOS in healthy volunteers, demonstrating good tolerability and confirming their prebiotic potential, as demonstrated by increased fecal bifidobacteria levels, increased short-chain fatty acid production, diminished fecal pH and reduction in 24h urinary excretion of *p*-cresol/PCS, which under steady state conditions is a proxy for the intestinal exposure [20–23]. Given the clinical data in healthy individuals and the preclinical data in an animal model of moderate CKD, the ‘AXOS in CKD study’ was designed to investigate the influence of AXOS in patients with CKD not yet on dialysis, thereby focusing on various microbiota derived uremic retention solutes and the occurrence of insulin resistance.

## Materials and Methods

### Study population

CKD patients with an estimated glomerular filtration rate (eGFR) between 15 and 45 ml/min/1.73 m^2^, followed at the nephrology outpatient clinic of the University Hospitals Leuven, Belgium, were enrolled in the study (clinicaltrials.gov NCT02141815). Eligible patients were 18 years or older and able to give written informed consent. Patients with known gastro-intestinal disease (i.e., inflammatory bowel disease, malignancy), previous colorectal surgery and insulin-dependent diabetes mellitus were excluded. Use of antibiotics, prebiotics or probiotics in the past 4 weeks was not allowed. The study was performed according to the Declaration of Helsinki and approved by the Ethics Committee (ML9503) of the University Hospitals Leuven. Written informed consent was obtained from all patients.

### Study design

**Fig 1:**
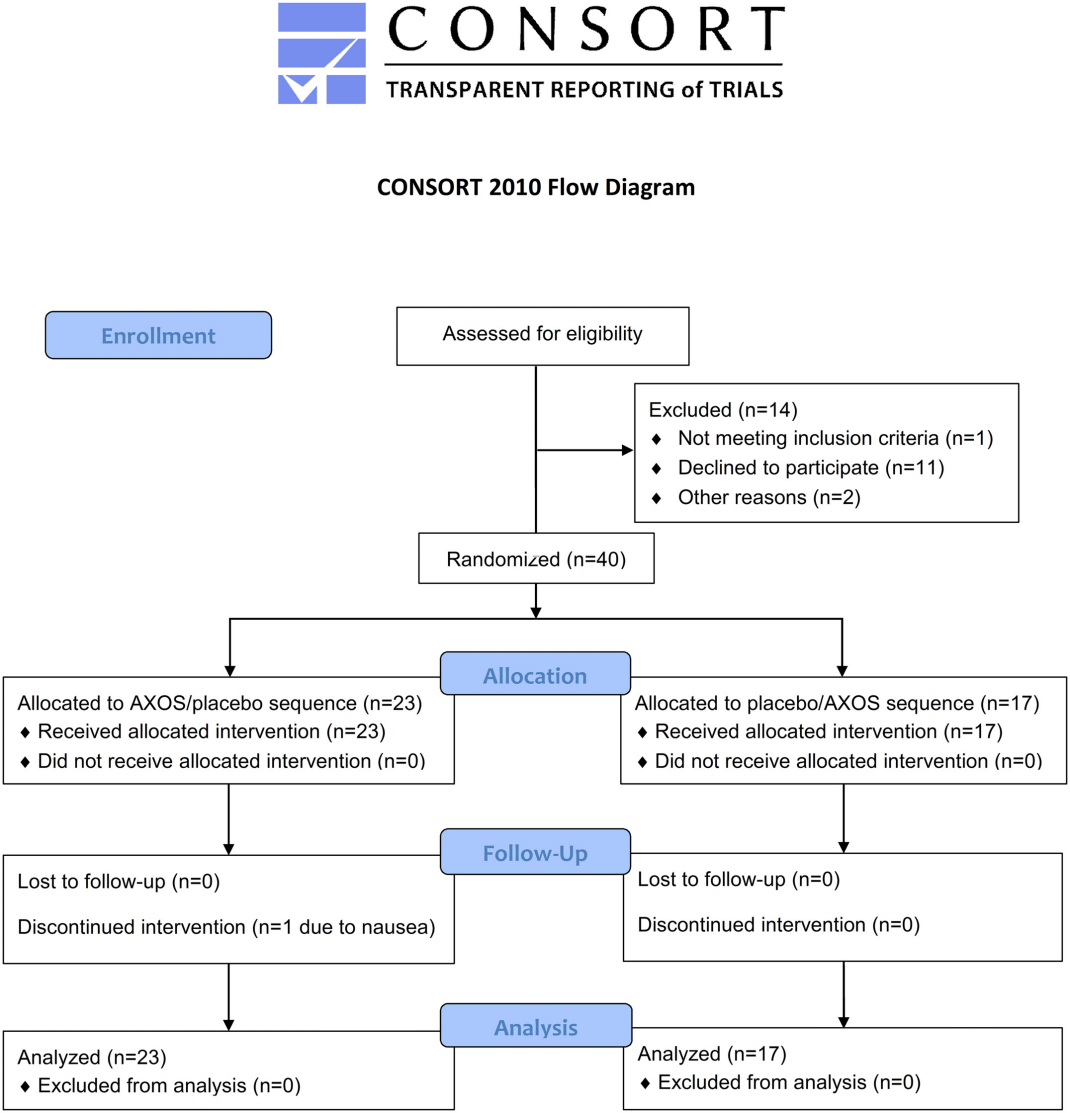
CONSORT flow diagram.

**Fig 2:**
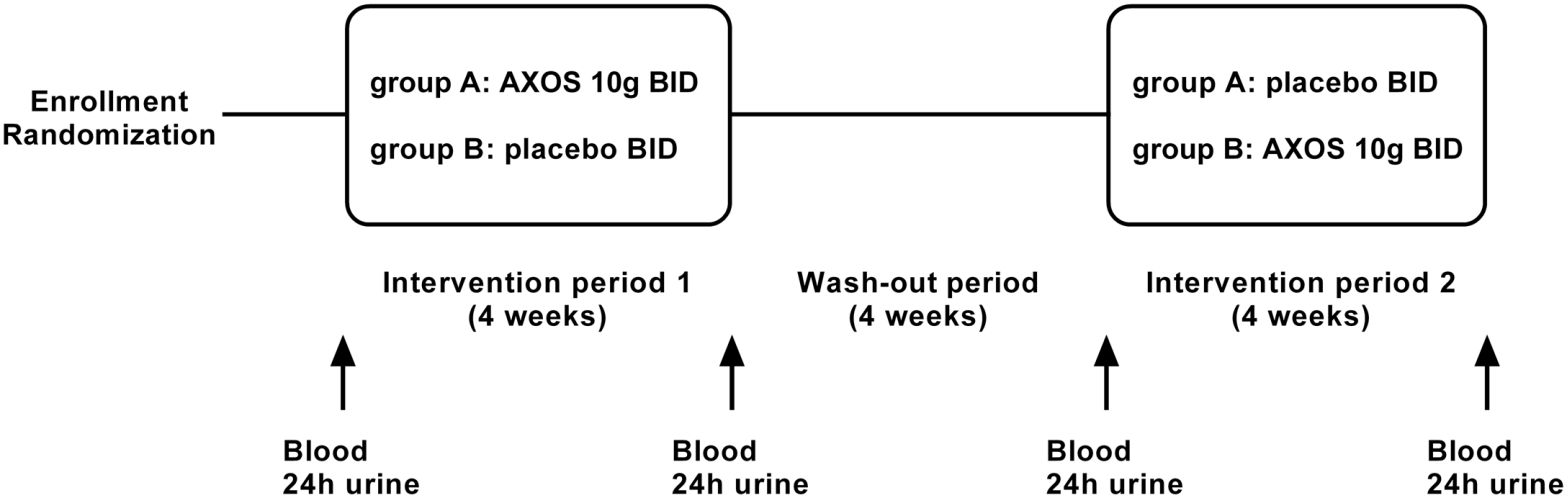
Study design. Schematic representation of the study design. AXOS, arabinoxylan oligosaccharides; BID, twice a day.

Figs 1 and 2 present the randomized, placebo-controlled, double-blind, cross-over study design (S1 CONSORT Checklist and S2 Protocol). After enrollment (performed by RP), patients were randomized by the study nurse, blinded from both the investigator and study participant. Randomization was performed by the sealed envelope system, in which the study nurse randomly opened a preformed envelope containing the allocated treatment regimen. Patients were allocated to sequential treatment with AXOS and placebo, or vice versa, with a wash-out period between both intervention periods. Treatment with AXOS or placebo was offered to patients in identical vials and boxes. Each box was also labeled with a numerical code, unique to treatment allocation and again blinded from both the investigator and study participant, as an additional measure to allow review of the correct treatment allocation by the study nurse. The duration of each intervention and wash-out period was 4 weeks with a total study duration of 12 weeks. At each time point, we collected blood (fasting), 24h urine, food frequency questionnaires (i.e., dietary habits during the past 4 weeks), and information regarding body weight, stool frequency and consistency (i.e., Bristol stool scale). After each intervention period, tolerance was assessed by five-level Likert item questionnaires for overall tolerance, study treatment intake, abdominal discomfort, abdominal cramps, abdominal bloating, flatulence, nausea and diarrhea. To evaluate safety, there was also clinical and additional biochemical (i.e., creatinine, C-reactive protein, alanine aminotransferase, potassium) follow-up at each time point.

### Study treatment

Active treatment consisted of AXOS 10 g twice daily, which were obtained from commercial wheat bran. The composition of AXOS and the procedures for their characterization are described elsewhere [24]. Maltodextrin (Paselli MD 6, Avebe, Veendam, The Netherlands), an oligosaccharide obtained by enzymatic hydrolysis of potato starch with total digestion in the small intestine, was used as placebo in equal volumes as AXOS (i.e., approximately 6.7 g twice daily). Both AXOS and maltodextrin were dissolved in 200 ml water prior to consumption.

### Biochemical measurements

At each time-point, blood (fasting) was taken by venous puncture for measurement of creatinine (mg/dl), urea (mg/dl), glucose (mg/dl), insulin (mU/l), C-reactive protein (mg/l), alanine aminotransferase (U/l), potassium (mmol/l), PCS (μM), PCG (μM), IS (μM), TMAO (μM) and PAG (μM). Creatinine (mg/dl), urea (mg/dl), glucose (mg/dl), insulin (mU/l), C-reactive protein (mg/l), alanine aminotransferase (U/l) and potassium (mmol/l) were measured using standard laboratory techniques. The eGFR was calculated using the Chronic Kidney Disease Epidemiology Collaboration (CKD-EPI) equation [25]. Insulin resistance was estimated by the homeostatic model assessment (HOMA-IR), which approximates the insulin—glucose product, divided by 405 [26]. Serum levels of PCS, PCG, IS, PAG and TMAO were quantified using a dedicated ultra-performance liquid chromatography—tandem mass spectrometry (UPLC—MS/MS) (Acquity—Xevo TQS, Waters, Zellik, Belgium) method. For sample preparation, 50 μl serum, 50 μl solution of MQ water/MeOH/0.01 N sodium hydroxide (% v/v 75/20/5), 20 μl of internal standard mixture (PCS-d7, PCG-d4, IS-d4, TMAO-d9 and PAG-d5) and 150 μl acetonitrile were thoroughly mixed in 96-well Ostro plates (Waters, Zellik, Belgium). Supernatants were collected, dried and after dilution with 1000 μl MQ water, injected on the UPLC-MS/MS system. Chromatographic separation was performed on an Acquity CSHFluoroPhenyl column (Waters, Zellik, Belgium). The mobile phase, delivered at a flow rate of 0.5 ml/min at 40°C, consisted of a gradient of 0.1% formic acid in MQ water (A) and MeOH (B). Ionization of PCS, PCG, IS, PAG and their corresponding isotopologues (internal standards) was achieved in negative mode, while ionization of TMAO and TMAO-d9 was performed in positive mode. The total, within-run, between-run and between-day method imprecision, according to the NCCLS EP5-T guideline, were below 10% for all compounds while their mean recoveries were between 96 and 101%. With availability of 24h urinary collections at each time point, we also measured 24h urinary excretion of urea, PCS, PCG, IS, TMAO and PAG. Assuming steady state conditions and negligible non-renal clearance, 24h urinary excretion can be considered to be an estimate of the daily intestinal uptake of microbial metabolites.

### Study endpoints

Primary efficacy endpoint was the effect of AXOS on serum levels of microbial metabolites. As secondary endpoints, we investigated both 24h urinary excretion of microbial metabolites and HOMA-IR. A sample size of 26 patients was expected to provide 80% power (two-sided, α = 0.05) for detection of a significant reduction of 10 μM in serum PCS. To anticipate potential drop-out, we aimed to include a total of 40 patients.

### Statistics

Data are expressed as mean (SD) for normally distributed variables or median (interquartile range [IQR]) for non-normally distributed variables. In an intention-to-treat analysis, treatment effect was tested using mixed models. For each variable, post-intervention value was considered the dependent variable with treatment, within-subject period and pre-intervention value entered as fixed effects into the model. Differences in post-intervention tolerance measurements (i.e., five-level Likert items) were evaluated by Fisher’s exact test. For all statistical analyses, *P*-values less than 0.05 were considered significant. All statistical analyses were performed using SAS (version 9.3, the SAS institute, Cary, NC).

## Results

### Study population

Between May 2014 and March 2015, a total of 40 patients were included in the study. Table 1 lists the baseline characteristics. Vascular (18 patients) and glomerular disease (8 patients) were the most prevalent underlying renal diseases. Seven patients were treated with oral antidiabetic drugs. During study, there were no significant differences in total calorie intake (*P* 0.95), intake of protein (*P* 0.57), carbohydrate (*P* 0.90), fiber (*P* 0.32) and fat (*P* 0.85), and body weight (*P* 0.13), when either AXOS or placebo was given as intervention.

**Table 1:** Study population.

Variabele	Mean (SD) or median (IQR)
Age (yr)	70 (6)
Gender: male/female (%)	28/12 (70/30)
Body mass index (kg/m^2^)	28.7 (5.0)
Creatinine (mg/dl)	1.98 (1.60–2.18)
eGFR (ml/min per 1.73 m^2^)	33 (27–38)
24h proteinuria (g)	0.161 (0.078–0.498)
Calories (Kcal/day)	1473.8 (412.1)
Protein (g/day)	57.7 (18.3)
Carbohydrate (g/day)	186.0 (54.8)
Fiber (g/day)	18.8 (7.5)
Fat (g/day)	52.4 (17.5)
Urea (mg/dl)	65.5 (51.0–75.5)
Serum *p*-cresyl sulfate (μM)	52.0 (25.5–76.5)
Serum *p*-cresyl glucuronide (μM)	0.18 (0.10–0.35)
Serum indoxyl sulfate (μM)	10.9 (7.6–15.3)
Serum trimethylamine N-oxide (μM)	8.9 (7.1–12.3)
Serum phenylacetylglutamine (μM)	6.5 (3.6–8.6)
Glucose (fasting) (mg/dl)	98 (93–104)
Insuline (fasting) (mU/l)	10.9 (7.3–16.7)
HOMA-IR	2.7 (1.7–4.4)

eGFR, estimated GFR; HOMA-IR; homeostatic homeostasis model assessment-estimated insulin resistance.

### Compliance and tolerance

Overall, compliance was excellent with intake of 96.6% of AXOS doses and 97.9% of placebo doses. One patient prematurely ended the study due to declared nausea during intervention with AXOS in the first period. In general, tolerance was good with no significant differences in overall tolerance (*P* 0.13), study treatment intake (*P* 0.07), abdominal discomfort (*P* 0.30), abdominal cramps (*P* 1.00), abdominal bloating (*P* 0.27), nausea (*P* 0.23) and diarrhea (*P* 1.00), when either AXOS or placebo was given as intervention. We, however, noted a significant change in flatulence (*P* < 0.001) with an increase in flatulence (i.e., Likert scale 4 and 5) in 30 versus 20 patients during intake of AXOS and placebo, respectively. Stool frequency (*P* 0.92) and consistency (*P* 0.62) were not significantly different between both interventions. Biochemically, there was no significant change in serum creatinine (*P* 0.91), C-reactive protein (*P* 0.37) and potassium (*P* 0.47), although we noted a small, albeit significant decrease in alanine aminotransferase (*P* 0.03).

### Serum levels and 24h urinary excretion of microbial metabolites

**Fig 3:**
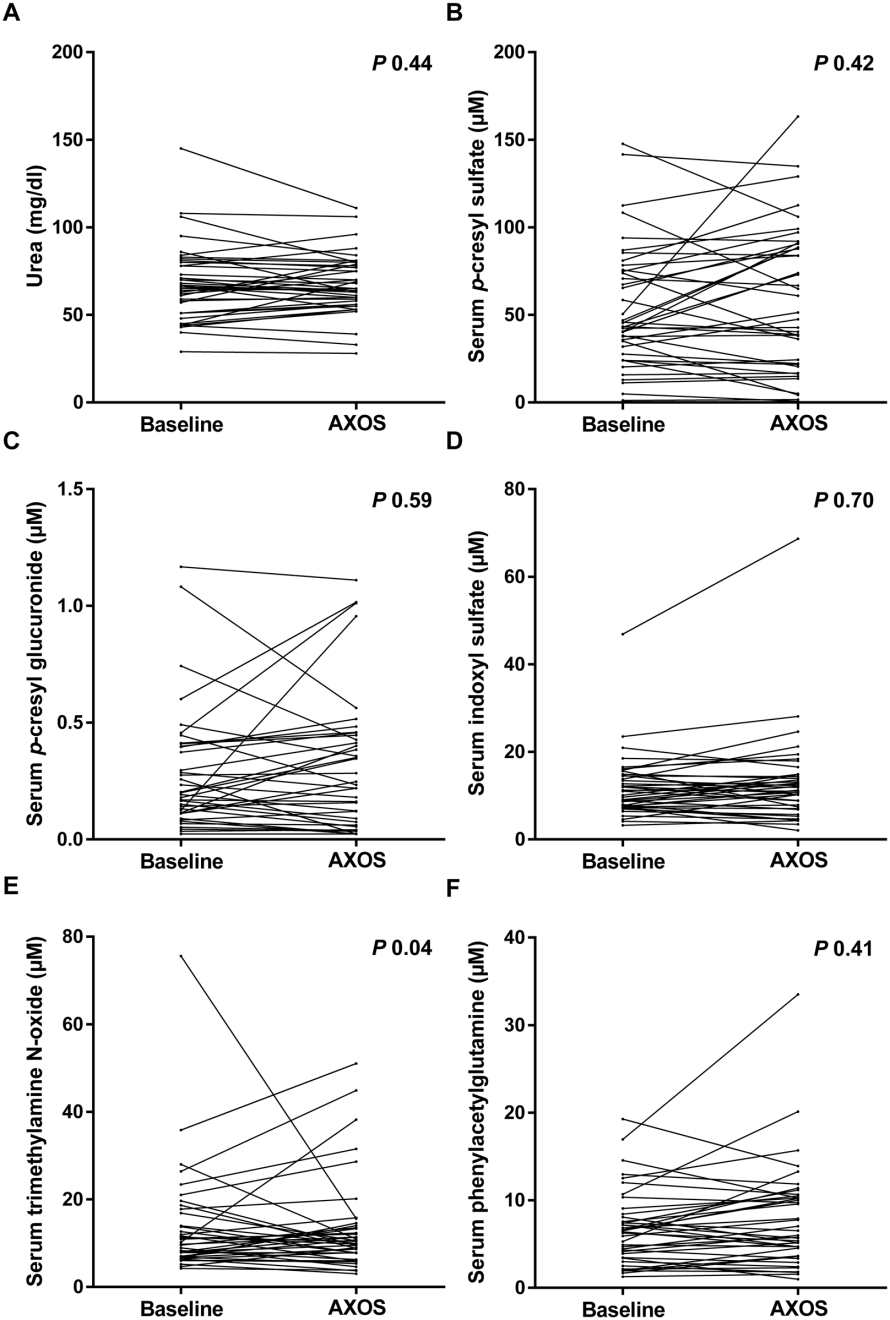
Serum levels of microbial metabolites during treatment with arabinoxylan oligosaccharides (AXOS). Evolution of serum urea (A), *p*-cresyl sulfate (B), *p*-cresyl glucuronide (C), indoxyl sulfate (D), trimethylamine N-oxide (E) and phenylacetylglutamine (F) during treatment with AXOS.

As primary endpoint, the influence of AXOS versus placebo on serum levels of microbial metabolites was explored (Table 2). We observed no significant change in serum PCS (*P* 0.42), PCG (*P* 0.59), IS (*P* 0.70), and PAG (*P* 0.41) (Fig 3). There was a small, albeit significant decreasing effect of AXOS on serum TMAO (*P* 0.04). No effect was noted on urea (*P* 0.44). As secondary endpoint, we also considered 24h urinary excretion of microbial metabolites with no significant change in 24h urinary excretion of PCS (*P* 0.31), PCG (*P* 0.23), IS (*P* 0.87) and PAG (*P* 0.43) (Table 3). In contrast to what was noted for serum TMAO, there was no effect of AXOS on 24h urinary excretion of TMAO (*P* 0.97). Again, we observed no significant effect on 24h urinary excretion of urea (*P* 0.08). When only taking into account patients with initial lower than median fiber intake, there were neither differences in serum levels nor in 24h urinary excretion of any of the microbial metabolites (data not shown).

**Table 2:** Influence of arabinoxylan oligosaccharides (AXOS) on serum levels of microbial metabolites.

Solute	Treatment effect (AXOS vs. placebo) (95% confidence interval)	*P*
Urea (Ln)	0.035 (- 0.056–0.127)	0.44
*p*-Cresyl sulfate (Ln)	- 0.115 (- 0.401–0.171)	0.42
*p*-Cresyl glucuronide (Ln)	- 0.105 (- 0.496–0.286)	0.59
Indoxyl sulfate (Ln)	- 0.031 (- 0.198–0.136)	0.70
Trimethylamine N-oxide (Ln)(Ln)(Ln)	- 0.237 (- 0.464–0.010)	0.04
Phenylacetylglutamine (Ln)	0.080 (- 0.115–0.275)	0.41

**Table 3:** Influence of arabinoxylan oligosaccharides (AXOS) on 24h urinary excretion of microbial metabolites.

Solute	Treatment effect (AXOS vs. placebo) (95% confidence interval)	*P*
24h urinary excretion of urea (Ln)	0.091 (- 0.012–0.194)	0.08
24h urinary excretion of *p*-cresyl sulfate (Ln)	- 0.128 (- 0.382–0.126)	0.31
24h urinary excretion of *p*-cresyl glucuronide (Ln)	- 0.291 (- 0.776–0.194)	0.23
24h urinary excretion of indoxyl sulfate (Ln)	- 0.015 (- 0.213–0.182)	0.87
24h urinary excretion of trimethylamine N-oxide (Ln)	- 0.007 (- 0.410–0.396)	0.97
24h urinary excretion of phenylacetylglutamine (Ln)	0.090 (- 0.137–0.318)	0.43

### Insulin resistance

As co-secondary endpoint in the study, we analyzed the influence of AXOS versus placebo on insulin resistance, as determined by HOMA-IR (fasting) (Table 4). We observed no significant change in HOMA-IR (*P* 0.93), neither in levels of insulin (*P* 0.87), nor in those of glucose (*P* 0.73). In subgroup analysis of patients with initial lower than median fiber intake, there were also no differences in any of the 3 parameters of insulin resistance (data not shown). No effect on HOMA-IR, insulin and glucose was noted when also excluding patients on oral antidiabetic drugs (data not shown).

**Table 4:** Influence of arabinoxylan oligosaccharides (AXOS) on insulin resistance.

Solute	Treatment effect (AXOS vs. placebo) (95% confidence interval)	*P*
HOMA-IR (Ln)	- 0.011 (- 0.259–0.237)	0.93
Insulin (fasting) (Ln)	- 0.020 (- 0.261–0.222)	0.87
Glucose (fasting) (Ln)	0.010 (- 0.049–0.070)	0.73

HOMA-IR; homeostatic homeostasis model assessment-estimated insulin resistance.

## Discussion

In the ‘AXOS in CKD study’, we explored the influence of prebiotic AXOS in patients with CKD not yet on dialysis. The key findings are as follows: (i) overall tolerance of AXOS is excellent; (ii) there is no effect of AXOS on serum levels and 24h urinary excretion of PCS, PCG, IS, TMAO and PAG as markers of microbiota derived uremic retention solutes; (iii) there is no effect of AXOS on insulin resistance.

The colonic microbial metabolism is a key contributor to the syndrome of uremia and various microbial metabolites, including PCS, PCG, IS, TMAO and PAG, have been related to adverse outcomes in patients with varying degrees of renal dysfunction [3–14]. Due to their colonic origin, there is an opportunity to target these solutes at their site of production, for example by means of prebiotics. In a pilot study in hemodialysis patients (*n* = 22, uncontrolled), we have earlier demonstrated a significant reduction in serum PCS, but not IS, after intake of a mixture of oligofructose-enriched inulin [17]. In another study in hemodialysis patients (*n* = 56, placebo-controlled), Sirich *et al*. noted that resistant starch significantly decreases serum IS and, possibly, also PCS [18]. It can, however, be hypothesized that prebiotic therapy would be more beneficial in patients at earlier stages of CKD. Indeed, the deleterious effects of these microbial metabolites may not yet have been established and, therefore, may possibly be prevented. To the best of our knowledge, only one small study investigated the effect of prebiotics (i.e., inulin) in patients with CKD not yet on dialysis (*n* = 13, placebo-controlled cross-over) [27]. The study demonstrated a significant decrease in serum *p*-cresol/PCS, while the effect on other microbial metabolites was not studied. We performed a randomized placebo-controlled cross-over study with AXOS, another prebiotic selected based on preclinical and clinical data, in 40 patients with eGFR between 15 and 45 ml/min/1.73 m^2^. We found no effect of AXOS on serum levels of PCS, PCG, IS and PAG, and only a borderline significant effect (not adjusted for multi-comparison) on serum levels of TMAO. There was no influence of AXOS on 24h urinary excretion of any of the microbial metabolites. These findings are at odds with previous observations in healthy volunteers which demonstrated a significant decrease in 24h urinary excretion of *p*-cresol/PCS as an estimate of the daily intestinal uptake [20–23]. It remains to be elucidated why AXOS exerts a beneficial effect in healthy volunteers, but not in patients with CKD not yet on dialysis. As alterations in diet during study could interfere with potential treatment effects, dietary changes were formally excluded. Compliance was also excellent. Although cumulative exposure to AXOS was higher in the current study, it can be hypothesized that the colonic microbial metabolism in CKD may be more resistant to short-term intake of AXOS. Our teams have previously shown a distinct colonic microbial metabolism in hemodialysis patients [28], but little is known about potential changes in patients at earlier stages of CKD, although it seems plausible that the shift in microbial metabolism is less pronounced than in hemodialysis patients. Therefore, as an effect of other types of prebiotics, i.e., inulin and resistant starch, has been demonstrated in hemodialysis patients [17;18], it can also be suggested that these prebiotics are more powerful than AXOS, although direct comparison in patients with varying degrees of renal dysfunction requires further study.

In addition, as experimental studies have suggested that AXOS may diminish CKD-related insulin resistance, whether or not due to a concomitant decline in serum PCS [19], we explored the potential effect of AXOS on HOMA-IR, which is commonly used to estimate insulin resistance. Again, we could not demonstrate a beneficial effect after intake of AXOS, possibly due to the lack of effect on serum PCS. Whether other types of prebiotics may affect insulin resistance in CKD remains to be explored.

There are limitations to our study. First, to explore the influence of AXOS on microbiota derived uremic retention solutes, we focused on PCS, PCG, IS, TMAO and PAG as the most important representatives of this group of solutes. We cannot exclude effects of AXOS on other microbial metabolites. Second, fecal samples were not collected, precluding direct statements about the influence of AXOS on colonic microbial metabolism. Third, HOMA-IR was chosen as the marker of insulin resistance, which may fail to detect more subtle changes in glucose homeostasis. As serial measurements of lipid parameters were also not available in our study, the potential beneficial influence of AXOS on dyslipidemia was not explored. Finally, our study population mainly consisted of patients of Caucasian origin. Care must be taken to extrapolate our data to other patient populations.

In conclusion, we could not demonstrate an influence of the prebiotic AXOS on microbiota derived uremic retention solutes and insulin resistance in patients with CKD not yet on dialysis. Further study is necessary to elucidate whether prebiotic therapy with other characteristics, higher cumulative exposure or in different patient populations may be of benefit.

## Supporting Information

S1 CONSORT Checklist CONSORT 2010 Checklist AXOS in CKD study. (DOC)

S1 Protocol Original protocol AXOS in CKD study. (DOCX)
