# Supplementary material for: The Influence of Prebiotic Arabinoxylan Oligosaccharides on Microbiota Derived Uremic Retention Solutes in Patients with Chronic Kidney Disease: A Randomized Controlled Trial
Source: PLoS One. 2016 Apr 21;11(4):e0153893. doi: 10.1371/journal.pone.0153893 (PMC4839737; doi:10.1371/journal.pone.0153893)
Supplement: S1 Protocol — (DOCX) [file pone.0153893.s002.docx]

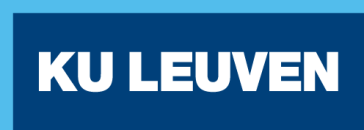

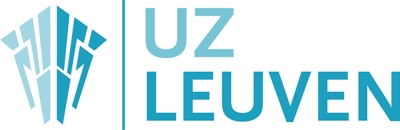


**PROTOCOL (ENGELS)**

**(version submitted to and approved by ethics committee before start of trial)**

**Titel studie (Engels):** The effect of arabinoxylan-oligosaccharides (AXOS) on intestinal generation of microbial metabolites in chronic kidney disease (S55578).

**Titel studie (Nederlands):** De invloed van arabinoxylaan-oligosacchariden (AXOS) op de darmaanmaak van microbiële metabolieten bij chronisch nierlijden (S55578).

**Studie type:** Academische, monocentrische studie

**Opdrachtgever onderzoek:** Ruben Poesen, MD (UZ Leuven)

Björn Meijers, MD PhD (UZ Leuven) Pieter Evenepoel, MD PhD (UZ Leuven)

Kristin Verbeke, Pharm PhD (KU Leuven)

**Summary:**

Chronic kidney disease is associated with the accumulation of various metabolites, i.e., uremic retention solutes. Evidence is mounting that the colonic microbiota contributes substantially to these uremic retention solutes. Indoxyl sulfate and *p*-cresyl sulfate are among the most extensively studied gut microbial metabolites, and are associated with cardiovascular disease, overall mortality and chronic kidney disease progression. The most important regulator of colonic bacterial metabolism is nutrient availability and especially the ratio of available fermentable carbohydrate to nitrogen, which can be modified by intake of so-called prebiotics (non-digestible food ingredients). Arabinoxylan oligosaccharides (AXOS) are a recently developed group of prebiotics, and already demonstrated a decreasing effect on intestinal generation of *p*-cresol in healthy individuals. Whether prebiotics in general, and AXOS more specifically, can influence intestinal generation of microbial metabolites in predialysis patients has not been studied to date. An interventional study with AXOS will therefore be initiated to test the hypothesis that AXOS can decrease intestinal generation and serum concentrations of microbial metabolites in patients with CKD not yet on dialysis.

**Introduction:**

Chronic kidney disease (CKD) profoundly disturbs the human metabolism, a disease state often referred to as uremia ^1^. Metabolites that are normally excreted by the kidneys accumulate when kidney function falls and eventually fails, i.e., they become so-called uremic retention solutes ^2^. Analyses show that the colonic microbiota contributes substantially to these uremic retention solutes, with indoxyl sulfate and *p*-cresyl sulfate being among the most discriminating biomarkers of uremia ^3^. Indoxyl sulfate is the end-product of bacterial proteolytic fermentation of the amino acid tryptophan to indole followed by endogenous oxidation and sulfate conjugation. Likewise, *p*-cresyl sulfate is the end-product of the combined actions of bacterial fermentation of tyrosine to *p*-cresol and endogenous sulfate conjugation ^4^. Observational studies in patients at various stages of CKD linked both indoxyl sulfate and *p*-cresyl sulfate to overall mortality ^5-7^, cardiovascular disease ^8;9^, and CKD progression ^10^. Further mechanistic studies demonstrated uremic concentrations of indoxyl sulfate and *p*-cresyl sulfate to elicit oxidative stress ^11;12^, to induce endothelial dysfunction ^13-15^ and cardiac remodeling ^16^, and to accelerate CKD progression ^17;18^.

The microbial metabolism is complex. One way the microbiota may be categorized is between saccharolytic (i.e., predominantly carbohydrate fermenters) versus proteolytic (i.e., predominantly protein fermenters) species. It is generally accepted that the most important regulator of bacterial metabolism is nutrient availability and especially the ratio of available carbohydrates to nitrogen ^19;20^. In case of carbohydrate excess, nitrogen will predominantly be incorporated in the expanding bacterial biomass. Conversely, in case of carbohydrate deprivation, mainly proteolytic fermentation will occur, thereby increasing the production of – among others – the indoles and cresols ^19;20^. Of note, it is suggested that CKD favors a proteolytic fermentation pattern with higher *p*-cresol generation rates in patients with renal dysfunction ^21^. Preliminary own findings also suggest that patients with CKD carry a different fecal microbial metabolite profile.

Prebiotics refer to ‘non-digestible food ingredients that beneficially affect the host by selectively stimulating growth or activity of a limited number of colonic bacteria and thus improve host health’ ^22;23^. At present, only bifidogenic, non-digestible oligo- and polysaccharides (particularly inulin, its hydrolysis product oligofructose and (trans)galacto-oligosaccharides) fulfill all criteria for prebiotic classification ^22;23^. We already demonstrated lowering of urinary *p*-cresol excretion (as a surrogate of intestinal generation) in healthy volunteers by ingestion of a mixture of inulin and fructo-oligosaccharides ^24^. In addition, we confirmed its potential in hemodialysis patients resulting in decreasing serum levels of *p*-cresyl sulfate ^25^. Wheat bran extract, a food-grade preparation highly enriched in arabinoxylan oligosaccharides (AXOS), is another recently developed prebiotic, which can reduce urinary *p*-cresol excretion in healthy volunteers ^26-28^. Interestingly, there is a clear difference between treatment responders and non-responders. Potential predictors of treatment efficacy are yet to be identified. In addition, whether prebiotics in general, and AXOS more specifically, can (partially) reverse the altered microbial metabolism and decrease intestinal generation of microbial metabolites in predialysis patients has not been studied to date. As these metabolites are related to CKD progression ^10^, prebiotics might prove a valuable adjuvant therapy in CKD to prevent or postpone progression to end-stage renal disease.

Therefore, an interventional study with AXOS will be initiated to test the hypothesis that prebiotics decrease intestinal generation of proteolytic metabolites in patients with CKD not yet on dialysis. The influence of AXOS on fecal metabolite profiles/metagenomics will be determined. Predictors of treatment response will be examined.

**Aims:**

- To explore the influence of AXOS on intestinal generation and serum concentrations of microbial metabolites (e.g., indoxyl sulfate and *p*-cresyl sulfate) in CKD
- To explore the influence of AXOS on fecal metabolite profiles/microbial composition in CKD
- To examine predictors of treatment response of AXOS in CKD

**Material and methods:**

***Study design***

12-week randomized interventional placebo-controlled cross-over study (see figure).


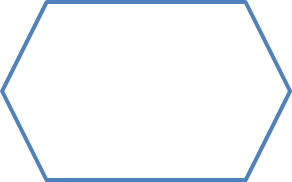

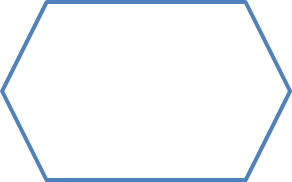


4 wk study period

4 wk study period

4 wk wash-out

Group A: AXOS 10g BID

Group B: AXOS 10g BID

Group A: placebo

Group B: placebo

Enrollement

Randomisation

***Study population***

Patients with chronic kidney disease will be recruited from the nephrology outpatient clinic at University Hospital Leuven. Patients will be instructed to minimize dietary habit changes during the treatment period. The primary efficacy endpoint is change in *p*-cresyl sulfate serum concentration after 4 weeks of treatment. Secondary endpoints include change in indoxyl sulfate serum concentration, change in *p*-cresyl sulfate generation rate (24h urinary excretion rate), change in indoxyl sulfate generation rate (24h urinary excretion rate), and change of fecal metabolite profile, each after 4 weeks of treatment. Based on preliminary data ^27^, a sample size of 40 patients is expected to provide 80% power for detection of a significant reduction of 10 µM in *p*-cresyl sulfate serum levels after 4 weeks of treatment (paired T-test, two-sided, α=0.05).

*Inclusion criteria:*

- Age ≥ 18 and ≤ 85 years
- Chronic kidney disease stage 3b-4, i.e., with estimated glomerular filtration rate (CKD-epi) between 45 – 15 ml/min/m² ^29^
- Written informed consent

*Exclusion criteria:*

- History of organic gastro-intestinal disease (e.g., inflammatory bowel disease, malignancy)
- History of colonic surgery
- Recipient of a renal or other solid organ transplant
- Use of pre-/pro-/syn- or antibiotics in preceding 4 weeks

***Intervention***

Active treatment period: AXOS 10g BID

Placebo period: maltodextrine

AXOS avDP4, av DAS 0.19 is registered as a food ingredient. Besides gastro-intestinal discomfort, so far no side-effects are documented ^26;27^. Subjective side-effects will be monitored during the study using symptom score systems. In addition, at each visit there will be a physical exam by a medical doctor. At baseline and after the last week of administration (week 12) there will be a routine lab during the scheduled nephrology consultation.

***Data collection***

See table

| **Procedures** | **Enrollment** | **Visit 1**  **(baseline)** | **Visit 2**  **(week 4)** | **Visit 3**  **(week 8)** | **Visit 4**  **(week 12)** |
| --- | --- | --- | --- | --- | --- |
| Informed consent | X |  |  |  |  |
| Medical and medication history | X |  |  |  |  |
| Physical exam+vital signs |  | x | x | x | x |
| Dietary history |  | x | x | x | x |
| Symptom score |  | x | x | x | x |
| Blood sample, fasting |  | x | x | x | x |
| 24h urine collection |  | x | x | x | x |
| Fecal sample: optional |  | x | x | x | x |

Blood sample (35ml): analysis of serum concentration of creatinine, urea, c-reactive protein, ALT, potassium, and microbial metabolites (e.g., indoxyl sulfate and *p*-cresyl sulfate)

24h urine collection: analysis of urinary concentration of creatinine, urea, and microbial metabolites (e.g., indoxyl sulfate and *p*-cresyl sulfate)

Fecal sample: analysis of fecal metabolite profile (GC-MS) and microbial composition (denaturing gradient gel electrophoresis, DGGE)

Reference List

(1) Meyer TW, Hostetter TH. Uremia. *N Engl J Med* 2007;357:1316-1325.

(2) Vanholder R, De SR. Pathophysiologic effects of uremic retention solutes. *J Am Soc Nephrol* 1999;10:1815-1823.

(3) Aronov PA, Luo FJ, Plummer NS et al. Colonic contribution to uremic solutes. *J Am Soc Nephrol* 2011;22:1769-1776.

(4) Meyer TW, Hostetter TH. Uremic solutes from colon microbes. *Kidney Int* 2012;81:949-954.

(5) Bammens B, Evenepoel P, Keuleers H, Verbeke K, Vanrenterghem Y. Free serum concentrations of the protein-bound retention solute p-cresol predict mortality in hemodialysis patients. *Kidney Int* 2006;69:1081-1087.

(6) Barreto FC, Barreto DV, Liabeuf S et al. Serum indoxyl sulfate is associated with vascular disease and mortality in chronic kidney disease patients. *Clin J Am Soc Nephrol* 2009;4:1551-1558.

(7) Liabeuf S, Barreto DV, Barreto FC et al. Free p-cresylsulphate is a predictor of mortality in patients at different stages of chronic kidney disease. *Nephrol Dial Transplant* 2010;25:1183-1191.

(8) Meijers BK, Claes K, Bammens B et al. p-Cresol and cardiovascular risk in mild-to-moderate kidney disease. *Clin J Am Soc Nephrol* 2010;5:1182-1189.

(9) Meijers BK, Bammens B, De MB, Verbeke K, Vanrenterghem Y, Evenepoel P. Free p-cresol is associated with cardiovascular disease in hemodialysis patients. *Kidney Int* 2008;73:1174-1180.

(10) Wu IW, Hsu KH, Lee CC et al. p-Cresyl sulphate and indoxyl sulphate predict progression of chronic kidney disease. *Nephrol Dial Transplant* 2011;26:938-947.

(11) Dou L, Jourde-Chiche N, Faure V et al. The uremic solute indoxyl sulfate induces oxidative stress in endothelial cells. *J Thromb Haemost* 2007;5:1302-1308.

(12) Motojima M, Hosokawa A, Yamato H, Muraki T, Yoshioka T. Uremic toxins of organic anions up-regulate PAI-1 expression by induction of NF-kappaB and free radical in proximal tubular cells. *Kidney Int* 2003;63:1671-1680.

(13) Dou L, Bertrand E, Cerini C et al. The uremic solutes p-cresol and indoxyl sulfate inhibit endothelial proliferation and wound repair. *Kidney Int* 2004;65:442-451.

(14) Meijers BK, Van KS, Verbeke K et al. The uremic retention solute p-cresyl sulfate and markers of endothelial damage. *Am J Kidney Dis* 2009;54:891-901.

(15) Tumur Z, Niwa T. Indoxyl sulfate inhibits nitric oxide production and cell viability by inducing oxidative stress in vascular endothelial cells. *Am J Nephrol* 2009;29:551-557.

(16) Lekawanvijit S, Adrahtas A, Kelly DJ, Kompa AR, Wang BH, Krum H. Does indoxyl sulfate, a uraemic toxin, have direct effects on cardiac fibroblasts and myocytes? *Eur Heart J* 2010;31:1771-1779.

(17) Gelasco AK, Raymond JR. Indoxyl sulfate induces complex redox alterations in mesangial cells. *Am J Physiol Renal Physiol* 2006;290:F1551-F1558.

(18) Miyazaki T, Ise M, Hirata M et al. Indoxyl sulfate stimulates renal synthesis of transforming growth factor-beta 1 and progression of renal failure. *Kidney Int Suppl* 1997;63:S211-S214.

(19) Birkett A, Muir J, Phillips J, Jones G, O'Dea K. Resistant starch lowers fecal concentrations of ammonia and phenols in humans. *Am J Clin Nutr* 1996;63:766-772.

(20) Smith EA, Macfarlane GT. Enumeration of human colonic bacteria producing phenolic and indolic compounds: effects of pH, carbohydrate availability and retention time on dissimilatory aromatic amino acid metabolism. *J Appl Bacteriol* 1996;81:288-302.

(21) Bammens B, Verbeke K, Vanrenterghem Y, Evenepoel P. Evidence for impaired assimilation of protein in chronic renal failure. *Kidney Int* 2003;64:2196-2203.

(22) Chow J. Probiotics and prebiotics: A brief overview. *J Ren Nutr* 2002;12:76-86.

(23) Roberfroid M, Gibson GR, Hoyles L et al. Prebiotic effects: metabolic and health benefits. *Br J Nutr* 2010;104 Suppl 2:S1-63.

(24) De P, V, Vanhoutte T, Huys G et al. Effects of Lactobacillus casei Shirota, Bifidobacterium breve, and oligofructose-enriched inulin on colonic nitrogen-protein metabolism in healthy humans. *Am J Physiol Gastrointest Liver Physiol* 2007;292:G358-G368.

(25) Meijers BK, De P, V, Verbeke K, Vanrenterghem Y, Evenepoel P. p-Cresyl sulfate serum concentrations in haemodialysis patients are reduced by the prebiotic oligofructose-enriched inulin. *Nephrol Dial Transplant* 2010;25:219-224.

(26) Cloetens L, Broekaert WF, Delaedt Y et al. Tolerance of arabinoxylan-oligosaccharides and their prebiotic activity in healthy subjects: a randomised, placebo-controlled cross-over study. *Br J Nutr* 2010;103:703-713.

(27) Francois IE, Lescroart O, Veraverbeke WS et al. Effects of a wheat bran extract containing arabinoxylan oligosaccharides on gastrointestinal health parameters in healthy adult human volunteers: a double-blind, randomised, placebo-controlled, cross-over trial. *Br J Nutr* 2012;1-14.

(28) Damen B, Cloetens L, Broekaert WF et al. Consumption of breads containing in situ-produced arabinoxylan oligosaccharides alters gastrointestinal effects in healthy volunteers. *J Nutr* 2012;142:470-477.

(29) Levey AS, Stevens LA, Schmid CH et al. A new equation to estimate glomerular filtration rate. *Ann Intern Med* 2009;150:604-612.
